# An Shen Ding Zhi Ling Ameliorates the Symptoms of Attention Deficit Hyperactivity Disorder via Modulating Brain-Derived Neurotrophic Factor-Related Signaling Pathways

**DOI:** 10.1155/2022/5471586

**Published:** 2022-07-21

**Authors:** Li Yaqun, Yuan Haixia, Song Yuchen, Zhu Mingxin, Lu Manqi, Tian Yunlong, Wang Aizhen, Han Xinmin

**Affiliations:** ^1^First Clinical Medical College, Nanjing University of Chinese Medicine, Nanjing, China; ^2^The Affiliate Taizhou Hospital of Nanjing University of Chinese Medicine, Taizhou, China; ^3^Jiangsu Key Laboratory of Pediatric Respiratory Disease, Nanjing, China; ^4^Jiangsu Province Hospital of Chinese Medicine, Nanjing, China; ^5^College of Traditional Chinese Medicine, Gansu University of Chinese Medicine, Lanzhou, Gansu, China

## Abstract

Attention deficit hyperactivity disorder (ADHD) is a common childhood neurodevelopmental disorder. It may impact the cognitive and social functions throughout childhood and determine adult outcomes. Dopamine (DA) deficiency theory is the pathogenesis of ADHD that is recognized by most international literature. Existing studies have shown that DA deficiency is caused by the abnormal function of the DA transporter and an imbalance in the DA receptor functionality. Recent clinical and experimental studies have found that the brain-derived neurotrophic factor (BDNF)/tropomyosin receptor kinase B (TrkB) signaling pathway acts a pivotal part in DA vesicle circulation and ADHD pathogenesis. An Shen Ding Zhi Ling (ASDZL) is a traditional Chinese medicine (TCM) prescription, which was widely prescribed to treat ADHD in Jiangsu, China, but its therapeutic mechanism is unclear. Therefore, we constructed a spontaneously hypertensive rat (SHR) model to explain its mechanism. SHRs were randomly assigned to four groups: SHR model group (vehicle), methylphenidate hydrochloride group (MPH), ASDZL group, and 7,8-dihydroxyflavone group (7,8-DHF). At the same time, the above groups were given continuous medication for four weeks. The results show that ASDZL, MPH, and 7,8-DHF group could significantly improve the spatial memory of SHRs in the Morris water maze tests. ASDZL increased the levels of BDNF, TrkB, p75 neurotrophin receptor (p75), C-Jun N-terminal kinases 1 (JNK1), and nuclear factor kappa B (NF-*κ*B) in the prefrontal cortex (PFC) and hippocampus synaptosome of SHRs. The results of this study suggest that ASDZL can relieve the symptoms of ADHD in SHRs by regulating the balance between the BDNF/TrkB signaling pathway (promoting vesicle circulation) and the BDNF/P75/JNK1/NF-*κ*B signaling pathway (inhibiting vesicle circulation) within the PFC and hippocampus synaptosome to increase the DA concentration in the synaptic cleft. The BDNF/TrkB signal pathway within the PFC and hippocampus synaptosome was activated by 7,8-DHF to increase DA concentration in the synaptic cleft. Whether 7,8-DHF can activate or inhibit the BDNF/P75 signaling pathway remains unclear.

## 1. Introduction

Attention deficit hyperactivity disorder (ADHD), as a common mental disorder in children, currently affects 5% of children around the world. Its symptoms are mainly inattention and hyperactivity-impulsivity [1]. Multivariate meta-regression analyses have shown that the pooled prevalence estimate of ADHD in China was 6.3% [2]. ADHD is a prevalent disorder, with the impairing condition beginning in early childhood and continuing into adolescence and adulthood. ADHD is frequently comorbid with other psychiatric disorders that can create substantial burdens for patients, their families, and communities [3].

The pathogenesis of ADHD remains unclear. Since Swanson et al. proposed the dopamine (DA) deficiency theory, which has been widely recognized and studied by the majority of the international academic community [4]. The DA system has become the focus of recent studies on the neurobiochemical pathogenesis of ADHD [5]. DA is an important modulator of learning and motivation [6] that performs a significant role in the pathogenesis of ADHD. The literature reports have demonstrated that DA deficiency is caused by the abnormal function of the dopamine transporter and an imbalance in the DA receptor function: these findings have formed a basic consensus in the academic community [7]. However, these conclusions do not fully explain the etiology and the pathogenesis of ADHD.

Numerous researchers have found that the BDNF/TrkB signaling pathway performs a crucial role in DA vesicle circulation and ADHD pathogenesis [8, 9]. Methylphenidate (MPH) is the first-line drug for the treatment of ADHD that can significantly improve the low activation state of the BDNF/TrkB signaling pathway [10].

Pro- and mature-BDNF activate two distinct receptors: p75 neurotrophin receptor (p75^NTR^) and TrkB [11]; therefore, the release of BDNF can also activate the P75 neurotrophic factor receptor, thereby activating JNK1 and NF-*κ*B, forming the opposite inhibition of the vesicle circulation pathway, inhibiting the sensitization of the AC/cAMP/PKA pathway, and affecting transmitter release [12–14].

Recently, there has been a significant increase in studies about the treatment of ADHD with traditional Chinese medicine (TCM). In the clinical syndrome definition, we propose that the positive pathogenesis of the disease is mostly “heart and liver fire, phlegm fire internal disturbance,” and “clearing the heart and calming the liver, eliminating phlegm and resuscitation, soothing the mind and calming the mind” as the treatment. ASDZL as an empirical formula of TCM has achieved positive clinical outcomes [15, 16]. Studies have shown that 7,8-DHF is a potent TrkB agonist, which can mimic BDNF and performs an important neuroprotective role in the dopaminergic neurons of mice [17].

In this study, our purpose was to explore whether ASDZL affects the function of the SNARE complex by regulating the BDNF/TrkB signaling pathway or downregulating the expression levels of the main factors (P75, JNK1, and NF-*κ*B) that inhibit the activation of related pathways and play a regulatory role in the initial process of vesicle circulation in the prefrontal cortex (PFC), corpus striatum, and hippocampus of ADHD model SHRs. In addition, we also assessed the capacity of the TrkB agonist 7,8-DHF in improving the core symptoms of ADHD after activating TrkB and the BDNF/P75 signal pathway.

## 2. Materials and Methods

### 2.1. Processing Method of ASDZL Decoction

ASDZL is composed of 12 Chinese herbs (Cu Chai Hu, Huang Qin, Lian Qiao, Yu Jin, Jue Ming Zi, Tian Zhu Huang, Gou Teng, Sheng Di Huang, Shi Chang Pu, Dang Gui, Yi Zhi Ren, and Yuan Zhi) [18]. All herbs were provided and authenticated by Professor Jianguo Chao. Twelve kinds of Chinese herbs were mixed with distilled water in a ratio of 1:8 and decocted twice for 2 hours each time [19]. Then the above decocting solution was further concentrated into 1.37 g/mL, and UHPLC was used to analyze the components of the aqueous extract of ASDZL [18].

### 2.2. Animal Experiments

Twelve WKY rats (3–4 weeks of age) and 48 SHRs (3–4 weeks of age) were purchased from Beijing Vital River Laboratory Animal Technology Co. Ltd. (Beijing, China, SCXK (Jing) 2016-0006) and maintained on a 12 h light/dark cycle with a temperature of 23 ± 2°C and relative humidity of 50%–60% in the experimental animal center of Nanjing University of Chinese Medicine. Animal experiments were performed in accordance with the National Institute of Health Guidelines for Laboratory Animals and were approved by the Animal Ethics Committee of Nanjing University of Chinese Medicine (No. 202010A039). The weight changes in rats of five groups are shown in Table 1.

### 2.3. Drug Administration

MPH is a commonly used drug in the clinical treatment of ADHD [20]. 7,8-DHF is a BDNF mimetic that acts as a TrkB receptor agonist [21, 22] and can be used to treat ADHD. MPH and 7,8-DHF were used as positive control drugs in this study. MPH was obtained from Janssen Cilag Manufacturing LLC (0AE008) and was mixed in 0.5% CMC-Na to a concentration of 0.1 mg/mL. 7,8-DHF was obtained from APExBIO (USA, B5484); it was first diluted with DMSO to 25 mg/mL and then diluted with PBS to a final drug concentration of 1 mg/mL. WKY rats were used as a normal control group, and the SHRs were randomly grouped into the following groups (*n* = 12): SHR group (model), MPH group (2 mg/kg/d), ASDZL group (27.4 g/kg/d), and 7,8-DHF group (5 mg/kg/d). As shown in Table 1, there was no significant difference in body weight among all groups. The WKY and SHR groups were orally administrated with 0.5% CMC-Na, whereas the MPH and ASDZL groups were given the corresponding drugs twice a day for four weeks. The 7,8-DHF group was intraperitoneally injected once a day. Dosages were calculated based on the average surface area of the bodies of humans and rats. Rats were weighed daily before administration, with gavage of 1.0 mL/100 g each time and injection of 0.5 mL/100 g each time according to their body weight. After the last behavioral test, the abdominal aorta of the rats was taken and sacrificed, and the plasma and brain tissues were stored at −80°C.

### 2.4. Preparation of Synaptosomes

The Percoll density gradient solution was prepared based on the concentration ratio within 30 min before the experiment. Fresh brain tissue was taken after the rats were killed. Impurities such as blood were removed with a large amount of freezing point homogenization buffer and blotted on filter paper before the tissue was weighed. The tissue was cut into a grinding tube according to the requirement of 1:9 (w/v). After centrifugation, the supernatant was taken and further diluted to 10–12 mL with freezing point homogenization buffer and stored on ice. The supernatant protein content was measured and diluted to 5–7 mg/mL. After dilution, 2 mL of supernatant per tube was spread evenly and slowly on a 3% gradient belt and centrifuged at 31876 × *g* for 5 min at 4°C; then the third and fourth layers of the gradient zone were removed, diluted with precooled sucrose/EDTA buffer, and centrifuged. Finally, the synaptosome was collected and fixed for 2 h in 2.5% glutaraldehyde for experimental use [23].

### 2.5. Behavioral Tests

After four weeks of treatment, the open field test (OFT) and Morris water maze (MWM) test were performed in each group [24, 25].

#### 2.5.1. OFT

The OFT is the most classic evaluation standard for the ADHD disease model [26]. The open-field arena was made of black-colored iron, whose total area (100 × 100 × 40 cm) was spaced into 16 squares with sides of 25 cm. The movement track of each rat was recorded every five minutes from 8:00 to 12:00, mainly including moving distance (m), time spent in the central area (s), and rearing times. The scent marks of each rat's urine and feces were removed with 75% ethanol between tests.

#### 2.5.2. MWM Test

The MWM test has been generally used to evaluate the learning and memory abilities of rats. The rats in each group were placed in MWM equipment with a water temperature of (24 ± 2°C) for testing, and the spatial acquisition trials, annulus visits, and time spent in the target quadrant (s) were collected.

### 2.6. Immunofluorescence

The tissues (PFC, corpus striatum, and hippocampus) of each group were sampled, fixed with 4% paraformaldehyde, then embedded in paraffin, and further cut into 3–4 *μ*m sections. The expressions of BDNF, P75, and NF-*κ*B in brain sections were measured by immunofluorescence. DAPI was used to restain the nuclei for immunofluorescence staining and microscopic observation. The average fluorescent intensity was analyzed by the Image-J software [25].

### 2.7. Western Blotting

The same amount of protein was extracted from each tissue and further separated by SDS-PAGE and transferred to the PVDF membrane. Anti-BDNF (1:1,000), TrkB (1:1,000), P75 (1:1,000), JNK1 (1:1,000), NF-*κ*B (1:1,000), and *β*-actin (1:2,000) were successively added and incubated overnight at 4°C. After incubation with secondary antibody, protein bands were quantitatively analyzed using ChemiDoc™ MP Imaging System HE Image Lab software.

### 2.8. Polymerase Chain Reaction (PCR)

Total RNA was isolated from the PFC, corpus striatum, and hippocampus tissues, and its purity and concentration were determined. All primers were designed and synthesized by Shanghai Shenggong Biological Engineering Co. Ltd. (Table 2). Five microliter SYBR Green master mix, 0.2 *μ*L reverse primers, and 1.0 *μ*L of cDNA (1:3 dilution) were mixed together to react. The dissociation curve of the reaction solution was measured to further evaluate the specificity of the amplified products. The melting curve and amplification plots of BDNF, TrkB, P75, JNK1, NF-*κ*B, and GAPDH in the PFC, corpus striatum, and hippocampus samples were recorded for all rats.

### 2.9. Statistical Analysis

All data were processed by GraphPad Prism 8.0 software.

## 3. Results

### 3.1. Effect of ASDZL on the Behavior of SHRs in the OFT

**Figure 1:**
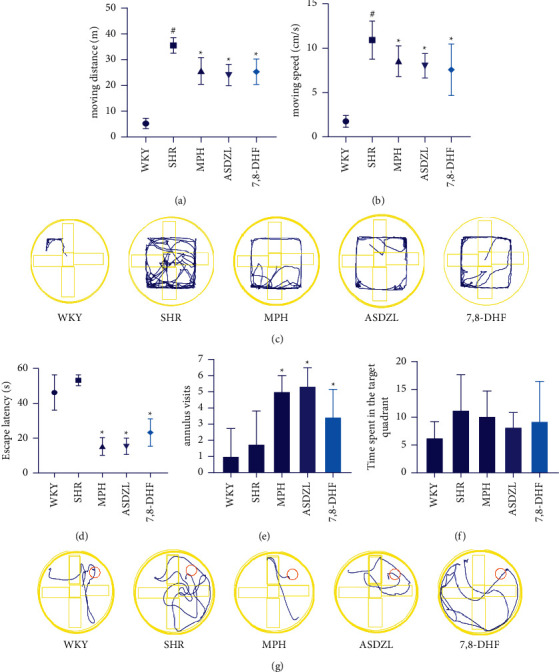
OFT and MWM test: (a) distance of movement (m) in OFT, (b) time spent (s) in OFT, (c) representative trajectories in OFT, (d) escape latencies in MWM test, (e) number of annulus visits in MWM test, (f) time spent (s) in the target quadrant in MWM test, and (g) representative trajectories in MWM test. *n* = 8 for each group. ^#^*p* < 0.05, SHR vs. WKY group. ^*∗*^*p* < 0.05, compared to the SHR group.

ASDZL and 7,8-DHF had no significant effect on body weight and developmental indexes in rats, as shown in Table 1. The results showed a significantly increased in distance and speed of movement among WKY and SHRs. After four weeks of treatment, the distance and speed of movement of MPH, ASDZL, and 7,8-DHF groups were significantly decreased than that of the SHR group (Figures 1(a) and 1(b)). The representative pathways of all groups are shown in Figure 1(c).

### 3.2. Effect of ASDZL on the Behavior of SHRs in the MWM Test

The time for locating the escape platform in MPH, ASDZL, and 7,8-DHF groups were less than the SHR group (Figure 1(d)). Compared with the WKY group, the SHR group played more visits to the annulus and spent more time in the target quadrant, but the difference was not statistically significant (Figure 1(e)). The number of visits to the annulus by MPH, ASDZL, and 7,8-DHF groups were much greater than that of the SHR group (Figure 1(e)), whereas the time spent by the MPH, ASDZL, and 7,8-DHF groups was less than that in the SHR group, with no significant difference (Figure 1(f)). The representative swimming patterns are shown in Figure 1(g).

### 3.3. Effect of ASDZL on BDNF, P75, NF-*κ*B in the PFC, Corpus Striatum, and Hippocampus (Immunofluorescence)

**Figure 2:**
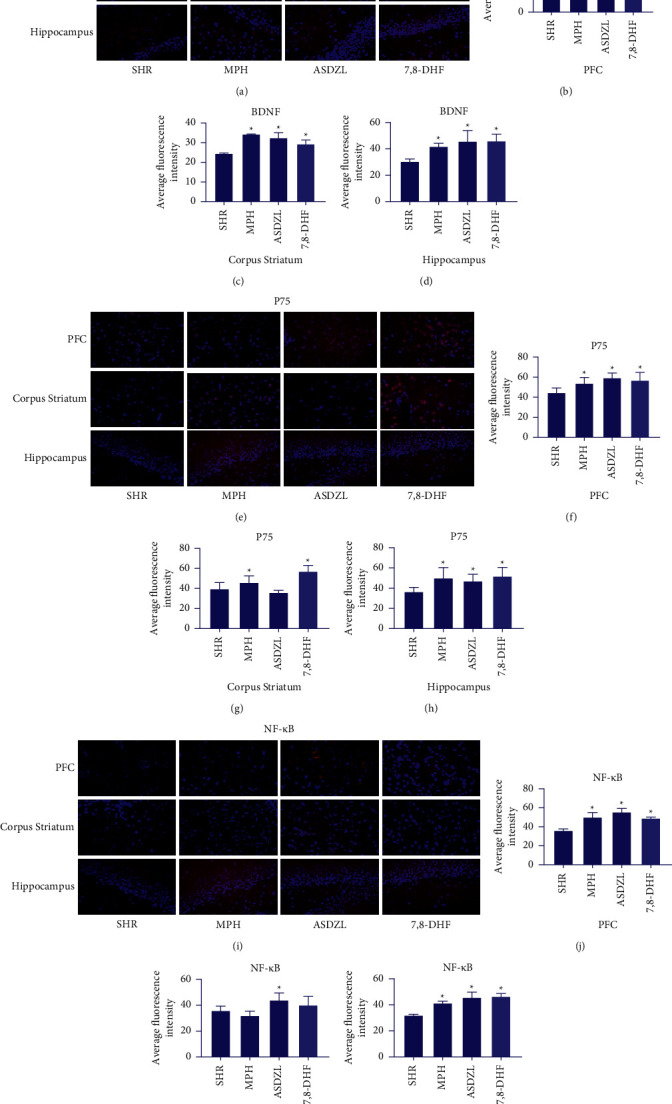
Effect of ASDZL on BDNF, P75, NF-*κ*B in PFC, corpus striatum, and hippocampus (immunofluorescence): (a) representative images of BDNF-immunopositive microglia (red) with DAPI (blue); average fluorescence intensity of BDNF in PFC (b), in corpus striatum (c), and in hippocampus (d). (e) Representative images of P75-immunopositive microglia (red) with DAPI (blue); average fluorescence intensity of P75 in PFC (f), in corpus striatum (g), and in hippocampus (h). (i) Representative images of NF-*κ*B-immunopositive microglia (red) with DAPI (blue); average fluorescence intensity of NF-*κ*B in PFC (j), in corpus striatum (k), and in hippocampus (l). *n* = 3 for each group. ^*∗*^*p* < 0.05, compared to the SHR group.

Higher average fluorescence intensities of BDNF (Figures 2(a), 2(b), and 2(d)), P75 (Figures 2(e), 2(f), and 2(h)), and NF-*κ*B (Figures 2(i), 2(j), and 2(l)) were showed in the PFC and hippocampus of the MPH, ASDZL, and 7,8-DHF groups compared to the SHR group. There was no significant discrepancy in fluorescence intensity of P75 in the corpus striatum between the ASDZL and SHR groups (Figures 2(e) and 2(g)) or NF-*κ*B among the MPH, 7,8-DHF, and SHR groups (Figures 2(i) and 2(k)). The MPH, ASDZL, and 7,8-DHF groups showed that the fluorescent intensity of BDNF was increasing significantly (Figures 2(a) and 2(c)).

### 3.4. Effect of ASDZL on the BDNF/TrkB Signaling Pathway in the PFC, Corpus Striatum, and Hippocampus (Western Blotting)

**Figure 3:**
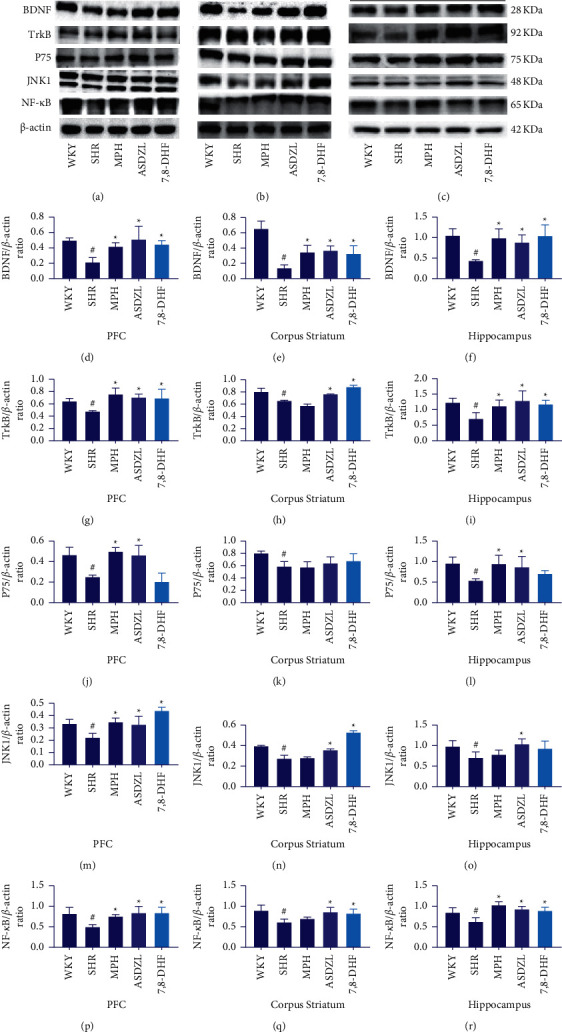
Effect of ASDZL on BDNF, TrkB, P75, JNK1, and NF-*κ*B in the PFC, corpus striatum, and hippocampus (western blotting): (a)–(c) western blot analysis of BDNF, TrkB, P75, JNK1, NF-*κ*B, and *β*-actin; (d)–(f) relative levels of BDNF; (g)–(i) relative levels of TrkB; (j)–(l) relative levels of P75; (m)–(o) relative levels of JNK1; and (p)–(r) relative levels of NF-*κ*B. *n* = 3 for each group. ^#^*p* < 0.05, SHR vs. WKY group. ^*∗*^*p* < 0.05, compared to the SHR group. The scale bar corresponds to 1.0 *μ*m.

To further observe whether ASDZL affects BDNF/TrkB signaling pathway, the PFC, corpus striatum, and hippocampus proteins were measured. Compare with the WKY group, the levels of BDNF and TrkB were remarkably lower in the SHR group. Following four weeks of treatment, the levels of BDNF and TrkB were significantly increased in the MPH, ASDZL, 7,8-DHF, and SHR groups, except for the level of TrkB in the corpus striatum of the MPH group (Figures 3(a)–3(i)).

### 3.5. Effect of ASDZL on the BDNF/P75/JNK1/NF-*κ*B Pathway in the PFC, Corpus Striatum, and Hippocampus (Western Blotting)

The total levels of relevant proteins were measured to reveal the effect of ASDZL on the BDNF/P75/JNK1/NF-*κ*B pathway in the PFC, corpus striatum, and hippocampus. The levels of P75, JNK1, and NF-*κ*B were conspicuously lower in the SHR group than in the WKY group. The level of P75 showed a conspicuously increase in the PFC and hippocampus of the MPH and ASDZL groups, while there was no conspicuous divergence in the corpus striatum of the MPH, ASDZL, 7,8-DHF, and SHR groups. ASDZL conspicuously increased the level of JNK1 in the PFC, corpus striatum, and hippocampus, whereas MPH only conspicuously increased the level of JNK1 in the PFC. 7,8-DHF aggrandized the level of JNK1 in the hippocampus. The NF-*κ*B/*β*-actin ratios of the PFC, corpus striatum, and hippocampus were significantly increased in the MPH, ASDZL, 7,8-DHF, and SHR groups, except for the level of NF-*κ*B level in the corpus striatum of the MPH group (Figure 3(a) and 3(j)–3(r)).

### 3.6. Effect of ASDZL on the BDNF/TrkB Pathway in the PFC, Corpus Striatum, and Hippocampus (qPCR)

**Figure 4:**
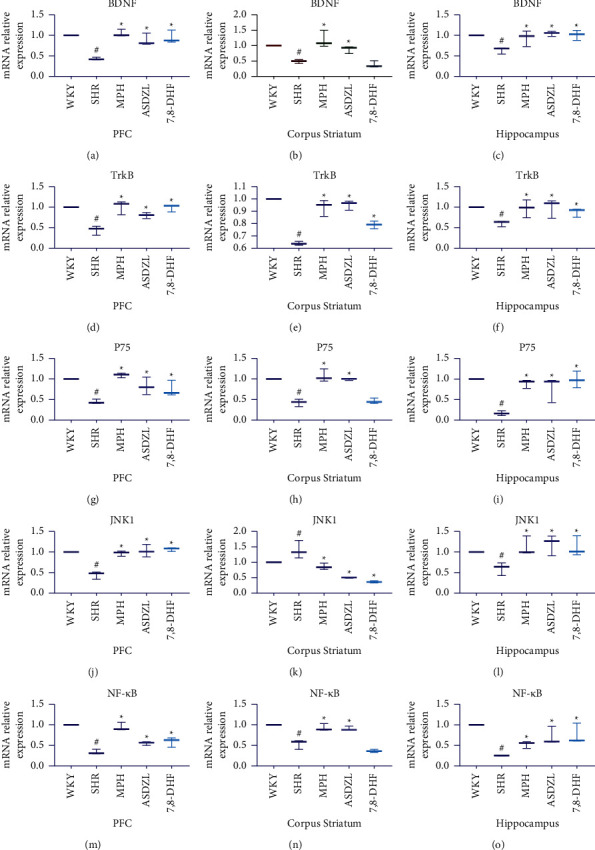
Effect of ASDZL on BDNF, TrkB, P75, JNK1, and NF-*κ*B in the PFC, corpus striatum, and hippocampus (qPCR): (a)–(c) relative expression of BDNF mRNA levels; (d)–(f) relative expression of TrkB mRNA levels; (g)–(i) relative expression of P75 mRNA levels; (j)–(l) relative expression of JNK1 mRNA levels; and (m)–(o) relative expression of NF-*κ*B mRNA levels. *n* = 3 for each group. ^#^*p* < 0.05, SHR vs. WKY group. ^*∗*^*p* < 0.05, compared to the SHR group.

To determine the effect of ASDZL on the BDNF/TrkB signaling pathway, we analyzed the relative expression of BDNF and TrkB mRNA in the synaptosomes of the PFC, corpus striatum, and hippocampus. The levels of BDNF and TrkB in the SHR group were conspicuously lower than those of the WKY group. After treatment, MPH and ASDZL conspicuously increased the expression of BDNF and TrkB in the PFC, corpus striatum, and hippocampus. However, the 7,8-DHF group displayed no conspicuously divergence in the BDNF of the corpus striatum compared to the SHR group (Figures 4(a)–4(f)).

### 3.7. Effect of ASDZL on the BDNF/P75/JNK1/NF-*κ*B Pathway in the PFC, Corpus Striatum, and Hippocampus

To understand the effect of ASDZL on the BDNF/P75/JNK1/NF-*κ*B pathway in the PFC, corpus striatum, and hippocampus, we also measured the relative expression levels of mRNA. The relative expression levels of P75 and NF-*κ*B were conspicuously lower in the SHR group than in the WKY group. However, the relative expression levels of JNK1 in the PFC and hippocampus of the SHR group were opposite to the expression trend in the corpus striatum. The MPH and ASDZL groups conspicuously increased the levels of P75, JNK1, and NF-*κ*B in the PFC, corpus striatum, and hippocampus; however, there was no conspicuous divergence in the levels of P75 and NF-*κ*B in the corpus striatum between the 7,8-DHF and SHR groups. MPH, ASDZL, and 7,8-DHF conspicuously decreased the expression of JNK1 in the corpus striatum compared with the SHR group (Figures 4(g)–4(o)).

## 4. Discussion

ADHD is characterized by the heterogeneity of attention deficiency, impulsiveness, hyperactivity, variable, and frequent comorbidities [27]. Although the evidence is lacking, it is generally recommended to use MPH, a central stimulant, to relieve ADHD in children [28]. The most common adverse events of methylphenidate are headache, loss of appetite, and insomnia [29]. Parents are often concerned about the side effects of these stimulants and tend to consider the use of complementary and alternative drugs, such as TCM to relieve the core symptoms of ADHD [30, 31]. ASDZL is a prescription of TCM created by Professor Han Xinmin and prescribed to relieve ADHD. Clinical researches have demonstrated that ASDZL could relieve symptoms of ADHD, especially the hyperactive-impulsive type [32]. In this study, we evaluated the role of ASDZL and 7,8-DHF on the behavioral performance and synaptic vesicle dopamine release mechanism in ADHD, which provided new synaptic-level views for treating ADHD.

Synaptosomes can mimic various aspects of synaptic functions, which are continually used as objects in neurobiology studies [33, 34]. The release of neurotransmitters is maintained through endocytosis and refilling of presynaptic local synaptic vesicles [35]. A previous study found that ASDZL significantly upregulated the expression of tyrosine hydroxylase and dopa decarboxylase, which are related to DA synthesis in the presynaptic membrane of SHR rats and increased the expression levels of synaptic-associated protein 25 and syntaxin-1A involved in the formation of SNARE complexes and vesicle-associated membrane proteins, among other protein and mRNA, thereby improving the core symptoms of ADHD [36]. The main active ingredients of ASDZL (Baicalin) can significantly increase the expression level of BDNF. Therefore, ASDZL has a special affinity for the DA system and potential regulatory effect on the DA synaptic vesicle cycle [37–40]. Synaptic-associated protein 25 and vesicle-associated membrane proteins are the key proteins constituting the SNARE complex and cooperate with syntaxin-1A and other proteins to participate in the key links of vesicle circulation such as DA vesicle anchoring, membrane transport, membrane fusion, and vesicle exocytosis in the form of SNARE complexes, as well as affect the brain. The level of internal DA regulates the learning and memory abilities of individuals [41–43]. A recent study showed that ASDZL regulated the activity of synaptosome ATPase and LDH enzymes in model rats and increased the content of AC, cAMP, and PKA in synapses, which suggested that ASDZL may perform a therapeutic role by reducing the inhibitory function of D2RS on the AC/cAMP/PKA signaling pathway [44].

Substantial evidence indicates that PFC can regulate attention and working memory by modulating memory-related activity within the PFC [45–47]. The striatum is richly innervated by midbrain DA neurons, which regulate a range of cellular and synaptic functions that control goal-directed behavior and habits [48, 49]. The hippocampus serves a key function in memory, navigation, and cognition [50]. Studies have shown that the three brain regions (PFC, corpus striatum, and hippocampus) are closely related to the onset of ADHD [51–53].

The DA system in the brain is a large and complex neural network. Further study will be required to determine the causes of defects in DA formation. The BDNF/TrkB signaling pathway might participate in regulating the DA synaptic vesicle circulation by affecting the protein receptor complex, which is closely related to ADHD pathogenesis.

In this study, we intended to examine the impacts of drugs on the BDNF-related pathways that influence the release of DA from synaptic vesicles in three regions of the brain and to determine whether drugs affect differently in different brain regions. Because of the higher levels of BDNF in brain tissue, BDNF and its receptor TrkB are widely distributed in the brain cortex, striatum, hippocampus, and other DA neurons. BDNF is involved in DA neuron synapse growth, transmitter release, cell survival, membrane transport, and synapse formation [54]. TrkB is a transmembrane protein with a high-affinity receptor for BDNF [55, 56], which can mediate most of its biological functions. The decreased expression of BDNF can cause significant changes in DA neuron synapse morphology and dysfunction, leading to a decrease in DA content in the brain. This impact may be related to the low activation of the BDNF/TrkB signaling pathway, which inhibits the function of the SNARE complex and affects presynaptic membrane DA vesicle circulation and transmitter release [57, 58]. The phospholipase C*γ* was recruited and activated by phosphorylation of Tyr816 residue for promoting neuronal survival and implicating neurite outgrowth and synaptic plasticity [59]. Numerous literature have demonstrated that BDNF participates in the development of PFC were upregulated by catalpol (a Chinese herbal medicine commonly used for ADHD) [25]. The present study showed that MPH, ASDZL, and 7,8-DHF could significantly increase the levels of BDNF/TrkB in PFC synaptosomes. Moreover, ASDZL can significantly increase the levels of BDNF/TrkB in the corpus striatum and hippocampus synaptosome. The results showed that the effect of MPH on the levels of TrkB and 7-8-DHF was inconsistent with the levels of BDNF by western blotting and qPCR analyses. Therefore, ASDZL can activate the BDNF/TrkB pathway in the PFC, corpus striatum, and hippocampus synaptosome, while MPH and 7,8-DHF only activate the BDNF/TrkB pathway in the PFC and hippocampus synaptosome, thereby promoting the formation of SNARE complexes. This complex is the core component in the fusion process of synaptic vesicles and participates in the exocytosis process of almost all secretory cells, eventually promoting DA release from synaptic vesicles [60]. However, it remains unclear whether 7,8-DHF can upregulate the levels of BDNF and MPH can upregulate the levels of TrkB in the corpus striatum synaptosome. We speculated that TrkB is activated to phosphorylate and promotes its insertion on the surface of DA neuron synaptosomes to form dimers, activating the BDNF/TrkB signaling pathway and then activating PI3k and promoting TrkB vesicle transport, further enhancing the localized BDNF/TrkB signal. This will promote the formation of the SNARE complex, accelerating its transportation, anchoring, membrane fusion, and other processes, and promoting the circulatory efflux of DA vesicles. Further research will be planned to examine this conjecture.

Another classical signaling pathway of BDNF is the BDNF/P75 signaling pathway. We suspected that this might also affect the release of DA from synaptic vesicles. The P75 neurotrophic factor receptor (P75NTR) is a transmembrane glycoprotein and a low-affinity receptor for BDNF, binding to neurotrophic factors in regulating various functions [61]. The death domain of P75NTR interacts with a large number of intracellular molecules and regulates different signaling cascades, including NF-*κ*B and JNK/caspase pathways in different cellular contexts [62, 63]. Our results suggested that ASDZL can activate the BDNF/P75/JNK1/NF-*κ*B signaling pathway in the PFC and hippocampus synaptosome. However, its effect on P75, JNK1, and NF-*κ*B within the corpus striatum synaptosome remains unclear, with the results for the expression levels of JNK1 were inconsistent between the western blot and qPCR analyses. The analyses showed that MPH could not significantly increase the level of JNK in the hippocampus synaptosomes compared to the model group by Western blotting. The effects of 7,8-DHF on the BDNF/P75/JNK1/NF-*κ*B signaling pathway in the synaptosome of the three brain regions were also not consistent. Therefore, our results suggested that ASDZL promoted the release of DA in the synaptic vesicles by launching the BDNF/TRKB pathway in the PFC, hippocampus synaptosome, and the BDNF/P75/JNK1/NF-*κ*B pathway to inhibit synapses. The release of DA in the vesicles that regulated the DA concentration in the synaptic cleft to reach a steady-state and achieved the goal of improving the core symptoms of ADHD. However, the effect of ASDZL on key factors (including pTrkB and PI3k that promote the enablement of the AC/cAMP/PKA signaling pathway and BDNF/TrkB signaling pathway in the synaptosome) is related to SNARE complex-mediated synaptic vesicle transport and anchoring links. The influence of the regulatory effects of factors and fusion link-related factors, among others, has not been studied.

## 5. Conclusion

The pathogenesis of ADHD is related to synaptic vesicle cycling. BDNF is an important factor that affects the synthesis, release, and clearance of dopamine in synaptic vesicles. The present study provided a preliminary explanation that the BDNF-related signaling pathway mechanism had an effect on the synthesis and release of DA in the synaptic vesicle cycle of ADHD. Although BDNF and its receptor TrkB are widely distributed in PFC, corpus striatum, hippocampus, and other areas rich in DA neurons, ASDZL only ameliorates the core symptoms of ADHD by adjusting the BDNF pathway in the PFC and hippocampus synaptosome. This mechanism is similar to the theory of balance between yin and yang in Chinese medicine. The 7,8-DHF could activate the BDNF/TrkB signal pathway in the PFC and hippocampus synaptosome to increase the concentration of DA in the synaptic cleft. Whether it can activate or inhibit the BDNF/P75 signaling pathway remains unclear and requires further study.

## Figures and Tables

**Table 1:** Weekly weight changes in rats of five groups.

Group	Day 0 (g)	Day 7 (g)	Day 14 (g)	Day 21 (g)	Day 28 (g)
WKY	68.2 ± 5.6	103.8 ± 6.7	135.8 ± 8.9	190.4 ± 17.0	210.7 ± 11.3
SHR	87.0 ± 5.5	118.7 ± 7.8	158.5 ± 11.9	197.9 ± 13.1	215.0 ± 14.6
MPH	100.9 ± 10.8	139.4 ± 10.7	183.9 ± 13.3	222.1 ± 12.1	232.0 ± 11.7
ASDZL	99.2 ± 10.6	130.4 ± 14.9	175.4 ± 17.7	213.2 ± 19.7	224.7 ± 19.7
7,8-DHF	100.3 ± 7.4	137.6 ± 8.9	181.1 ± 7.5	220.9 ± 9.8	231.7 ± 12.7

**Table 2:** Gene sequences of primers.

Genes	Primer length (kb)	Gene sequence	
GAPDH	20	F	GACATGCCGCCTGGAGAAAC
	20	R	AGCCCAGGATGCCCTTTAGT

BDNF	23	F	TGGTCAGTGGCTGGCTCTCATAC
	27	R	TCAGAACAGAACAGAACAGAACAGGAC

TrkB	23	F	GGTCTATGCCGTGGTGGTGATTG
	24	R	ATGTCTCGCCAACTTGAGCAGAAG

P75	23	F	CCTGCTGCTGCTGCTGATTCTAG
	20	R	CCACGCCTTCGCCCAAGTTG

JNK1	23	F	CACAGTGAGCAGAGCAGGCATAG
	25	R	TTGTCAGGAGCAGCACCATTCTTAC

NF-*κ*B	26	F	GATGGCTTCTATGAGGCTGAACTCTG
	23	R	CTTGCTCCAGGTCTCGCTTCTTC

## Data Availability

The original contributions presented in this study are included in the article; further inquiries can be directed to the corresponding authors.
